# The association between the Glutathione S-transferase polymorphisms and addiction to opioids and methamphetamine in the Iranian population

**DOI:** 10.3389/fpsyt.2024.1398666

**Published:** 2024-12-20

**Authors:** Mohammad Reza Eskandarion, Simin Jafaripour, Farid Heidari, Elham Talebi, Maryam Rezakhani Taleghani, Neda Maserat, Maryam Forutan, Raheb Ghorbani, Jaber Gharehdaghi, Reza Shirkoohi, Reza Raoofian

**Affiliations:** ^1^ Legal Medicine Research Center, Legal Medicine Organization, Tehran, Iran; ^2^ Cancer Research Institute, Imam Khomeini Hospital Complex, Tehran University of Medical Sciences, Tehran, Iran; ^3^ Department of Medical Genetics, School of Medicine, Mashhad University of Medical Sciences, Mashhad, Iran; ^4^ Department of Psychiatry, Semnan University of Medical Sciences, Semnan, Iran; ^5^ Department of Genetics, Faculty of Advanced Science and Technology, Tehran Medical Sciences, Islamic Azad University, Tehran, Iran; ^6^ Department of Genetics, Faculty of Biological Sciences, Shahrekord University, Shahrekord, Iran; ^7^ Department of Biology, Sistan and Balouchestan University, Zahedan, Iran; ^8^ Department of Biology, Islamic Azad University, Tehran, Iran; ^9^ Social Determinants of Health Research Center, Semnan University of Medical Science, Semnan, Iran

**Keywords:** Glutathione S-transferase, morphine, methamphetamine, substance use disorders, polymorphism

## Abstract

**Introduction:**

Glutathione S-transferase (GST) has the ability to detoxify the cellular environment of xenobiotic compounds and by-products of oxidative stress. The expression levels of GST genes and their polymorphisms are associated with various human diseases. Methamphetamine and opiate addiction also account for a significant proportion of SUDs in Iran. Considering the oxidative stress induced by morphine and methamphetamine and the potential of GST as a therapeutic option for SUD, we aimed to investigate the association of common genetic variations of two genes from GST family, GSTT1 and GSTM1, with addiction to morphine and METH in Iranian population.

**Material and methods:**

A total of 160 blood and urine samples were randomly collected from 50 opiums and 30 methamphetamine users and 80 healthy controls. All samples were processed by thin layer chromatography (TLC), high performance liquid chromatography, and Gas Chromatography-Mass Spectrometry (GC-MS) techniques to detect opium alkaloids (morphine, codeine, papaverine, noscapine, etc.), methamphetamine stimulants, and other legal and illegal drugs. The genotypes of GSTM1 and GSTT1 polymorphisms were determined by PCR. Statistical analysis was performed using the SPSS. This project was approved by the Research Ethics Committee of Legal Medicine Organization, Tehran, Iran.

**Results:**

A statistically significant association was observed between the GSTM1 polymorphisms and morphine addiction under a recessive genetic model. The reference group consisted of pooled n/p and p/p genotypes, with an odds ratio (OR) of 2.15, a 95% confidence interval (CI) of 1.05 to 4.39, and a P-value of 0.03. In contrast, there was no statistically significant association between genetic variations in the GSTT1 gene and morphine or methamphetamine addiction. The results revealed no significant association between GSTT1 and GSTM1 allele frequencies and morphine and methamphetamine addiction when divided into risk allele carriers and noncarriers.

**Conclusion:**

These findings suggest that the GSTM1 gene may be involved in the development of morphine addiction. However, further studies with larger sample sizes are required to verify these results and investigate the underlying molecular mechanisms.

## 1 Introduction

According to the most recent statistics published in World Drug Report, in 2021, approximately 36.3 million people (ranging from 19.6 to 53 million) are estimated to suffer from drug or substance use disorders (SUDs) all over the world (1, 2). SUDs and addiction are associated with higher risk of premature death, development of psychiatric conditions like depression and anxiety (3, 4), and transmission of viral infectious diseases such as acquired immunodeficiency syndrome (AIDS) and Hepatitis C (5).

Indeed, the etiology of SUDs and addiction is complex and modulated by a wide variety of factors, ranging from individual genetic make-up and exposure to environmental and emotional stress to personality and environmental factors like socioeconomic status and having access to drugs (6).

Regardless of the type of the substance of choice, all addictive substances are common in driving reward systems of brain through raising dopamine levels and altering brain’s function (7) though increased dopamine levels result from the activation of various molecular pathways which can vary depending on the specific class of addictive agent (8). Interestingly, a substantial body of genetics research studies have shown that SUDs are among the most heritable psychiatric diseases (8).

The heritability of SUDs ranges from 39-72% (6), with the highest estimates being for the most addictive substances (cocaine and opiates) (6) and for risk for progression from occasional use to dependence and addiction stages (6). SUDs follow complex pattern of inheritance and are controlled by multiple genes and their interaction with behavioural and environmental factors (8). It seems that the genetic factor contributes to the inheritance of variations in the pathways in which addictive substances exert their specific effects on the brain function (7). Accordingly, there are also some substance-specific genetic components despite the involvement of some shared genetic risk factors in the etiology of addiction to all drugs and alcohol (9, 10).

Methamphetamine and opiate addiction also comprise considerable percent of SUDs in Iran (11). Due to their prevalence in Iran, the present study focused on better understanding of genetic background of addiction to methamphetamine and opiate.

From biological standpoint, Methamphetamine (METH) exposure alters dopaminergic (12) and stereogenic systems (13). Therefore, METH exposure can cause dopaminergic terminal damage and excessive release of dopamine (DA) from neurons (13). Auto-oxidation of released DA leads to a higher production of free radicals of oxygen and nitrogen; the subsequent oxidative stress then mediates the activation of diverse neural cell apoptosis pathways (14).

The mechanism of action of morphine, as an agonist for G-protein coupled opioid receptors, almost involves binding to those receptors, induction of receptor conformational changes (15), and initiation/inhibition of diverse downstream signal transduction cascades involved in morphine activity and associated adverse effects (16, 17). However, same as METH, morphine stimulation also induces excessive generation of reactive oxygen and nitrogen species (RONS) and oxidative stress (OS) (18, 19) and also neuro inflammation (20).

Both METH and morphine which are known to introduce cellular reduction and oxidation (redox) imbalance are potentially able to negatively affect protein post-translational modifications (PTMs) which are sensitive to redox homeostasis (21). Amongst the most important redox-mediated PTMs on proteins following exposure to RONS are modifications on cysteine residues namely S-glutathionylation (22), where the reduced form of glutathione (GSH) is conjugated to low pK_a_ cysteine residues (22) and leads to change in the structure and function of the target proteins (22). This conjugation is catalyzed by glutathione S-transferases (GSTs), a superfamily of phase II metabolic isozymes which are best known for their ability to detoxify cellular environment from xenobiotic compounds as well as by-products of oxidative stress (23). Thus, given the extent of influences caused by GSTs in the cell following oxidative stress, it is unsurprising that the expression levels of GST genes and their polymorphisms are associated with a wide range of human diseases like diabetes and neurodegenerative diseases where oxidative stress is the central common factor (24). This study investigates the association between common genetic variations in two GST genes, GSTT1 and GSTM1, and addiction to morphine and METH in Iranian population and the potential role of GSTs in addressing SUDs, focusing on the oxidative stress induced by morphine and methamphetamine and the therapeutic potential of GSTs.

## 2 Materials and methods

### 2.1 Participants

A total of 160 blood samples were randomly selected from 50 opium users alive and 30 amphetamine user that were from Tehran Forensic Toxicology Laboratory and 80 healthy people as a control group. All subjects were of Iranian origin. This project was approved by the Research Ethics Committee of Legal Medicine Organization, Tehran, Iran. Inclusion criteria for the case group were: 1) Male participants; 2) History of opioid and/or amphetamine abuse, defined as a minimum of 6 months of continuous use; 3) Positive urine test result for opioids and/or amphetamines at the time of screening; 4) Negative results for heavy metal toxicity in the urine sample tested. All heavy metal levels, including lead, mercury, and arsenic, must be within the normal range and not indicative of toxicity; 5)Willing and able to provide written informed consent and comply with study procedures.

Healthy male individuals aged 18-65 years were considered eligible as controls if they had no current or past history of substance abuse or dependence, as determined by a structured clinical interview. Exclusion criteria for controls included: 1) Positive urine test result for opioids and/or amphetamines at the time of screening; 2) History of treatment for opioid and/or amphetamine addiction within the past 6 months; 3) History of current or past psychotic symptoms or a diagnosis of schizophrenia; 4) History of a serious medical condition that would interfere with study participation; 5) Positive urine test result for heavy metal toxicity at the time of screening; 6) Actively suicidal or a history of suicide attempts within the past year.

### 2.2 Laboratory measurements

Urine samples were collected from all participants and stored at 4°C until analysis. The samples were analyzed for the presence of opiates using Thin Layer Chromatography (TLC) techniques and were subjected to acid hydrolysis to cleave any drug conjugates and increase the detectability of the opiates. The narcotic drugs and amphetamine in the urine samples were then analyzed using gas chromatography-mass spectrometry (GC-MS) and high-performance liquid chromatography (HPLC) techniques. Dispersive liquid-liquid extraction (DLLE) methods and liquid-liquid extraction (LLE) were used for the extraction and detection of amphetamine (AMP), methamphetamine (METH), and other psychoactive substances. After urine extraction, the samples were analyzed using gas chromatography/mass spectrometry (GC/MS, Scion, 30 mm 0.250 m. 0.25 m, SS) and HPLC with a KNAUER (KNAUER GmbH, Berlin, Germany) C18 column (250 mm 4.6 mm, particle size: 5 m).

*For Heavy Metal Toxicity Test:* The urine samples were also analyzed for heavy metal toxicity, including lead, mercury, and arsenic. The samples were subjected to inductively coupled plasma mass spectrometry (ICP-MS) to determine the levels of these toxic metals in the urine.

All laboratory measurements were performed in accordance with standard operating procedures, and quality control measures were in place to ensure the accuracy and reliability of the results. The laboratory analyses were used to determine the presence of opiates and levels of heavy metal toxicity in the participants, which were then used to guide further evaluations and categorizations as needed.

### 2.3 DNA extraction and genotyping

3-5 ml blood samples were collected from all participants using standardized venipuncture techniques. DNA extraction was performed using the PrimePrep Genomic DNA Isolation Kit according to the manufacturer’s instructions. The concentration of the extracted DNA was determined using a NanoDrop spectrophotometer. The DNA purity was also assessed by calculating the ratio of absorbance from 260 nm to 280 nm. DNA samples with a 1.8-2.0 ratio were considered pure and used for further analyses.

Primers for GSTT1 and GSTM1 were designed using online Primer3 software. The primers were checked for specificity and synthesized by Pishgam Biotechnology Company. The following primer sequences were incorporated for differentiation between null and present alleles (**Table 1**). To ensure the quality of PCR reactions, β-globin primers were also designed and used as a positive control.

**Table 1:** The forward and reverse primer sequences for GST.

Gene name	Size (bp)	Primer sequence
GSTM1	132	F: GAACTCCCTGAAAAGCTAAAGC R: CTTGGGCTCAAATATACGGTGG
GSTT1	255	F: TTCCTTACTGGTCCTCACATCTC R: TCACCGGATCATGGCCAGCA
β-globin	273	F: CAACTTCATCCACGTTCACC R: GAAGAGCCAAGGACAGGTAC

The absence of β-globin PCR product in any sample was considered a technical failure, and those samples were excluded from further analyses. Negative controls were also included in each run to ensure the accuracy and reproducibility of the PCR reactions.

PCR amplification was performed in a final volume of 25 μl. The reaction mixture included 4 mM MgCl2, 2 mM dNTP, 1X PCR buffer, 0.25 U/ml polymerase enzyme, and 10 mM of both the forward and reverse primers, as final concentration. The Eppendorf™ Mastercycler X50 thermal cycler was programmed for an initial denaturation step of 5 min at 95°C, followed by 30 cycles of denaturation at 95°C for 1 min, annealing at 61°C for 1 min, and extension at 72°C for 1 min. A final extension step was performed at 72°C for 10 min. The amplified products were separated by gel electrophoresis to confirm the successful amplification of the target DNA. Gel electrophoresis was performed using a 2% agarose gel in TAE buffer. The amplified DNA products were loaded onto the gel and subjected to electrophoresis. All procedures were performed in accordance with standard operating procedures, and appropriate quality control measures were in place to ensure the accuracy and reliability of the results. The results of the DNA extraction, concentration measurement, primer design, PCR, and gel electrophoresis were used to confirm the presence of the target DNA in the participants and to guide further analyses as needed.

The n/p and p/p alleles in GSTM1 and GSTT1 were detected using PCR, with p/p in GSTM1 detected by a 132-bp fragment, while a co-amplified 273-bp fragment of the human myoglobin gene served as an internal standard. n/p homozygous deletion (null genotype) in GSTM1 was identified on the basis of presence of the 273-bp fragment of the human myoglobin gene band and absence of the 132-bp band.

p/p in GSTT1 was detected by amplifying a 255-bp fragment and co-amplifying a 273-bp fragment of the human b-globin gene using primers for the internal standard. n/p homozygous deletion (null genotype) was identified on the basis of the presence of the 273-bp band of b-globin gene and absence of the 255-bp band.

### 2.4 Statistical analysis

All statistical analyses were performed using the SPSS for Windows version 11.5 (SPSS Inc., Chicago, IL, USA) and STATA version 15.0 software (Stata Corp LLC, College Station, TX, USA). Due to the small sample size, Firth-type logistic regression analysis was selected to test the association of addiction to either morphine or methamphetamine and the genetic variant under recessive genetic model. By incorporating a term that counteracts the first-order term in the asymptotic expansion of the bias of the maximum likelihood estimation, the firth logistic regression essentially introduces a more effective score function. As the sample size grows, the term diminishes to zero (Firth, 1993; Heinze and Schemper, 2002). Firth’s method is comparable to penalizing the likelihood by the Jeffreys invariant prior for generalized linear models with canonical linkages, like logistic regression. This approach is appealing because it reduces bias for small sample sizes and produces reliable, finite estimates even when there is separation. Also, both variants were tested for deviations from the Hardy–Weinberg equilibrium via the X2 test with two degrees of freedom in both case and control groups, and no deviation was found.

## 3 Results

**Figure 1:**
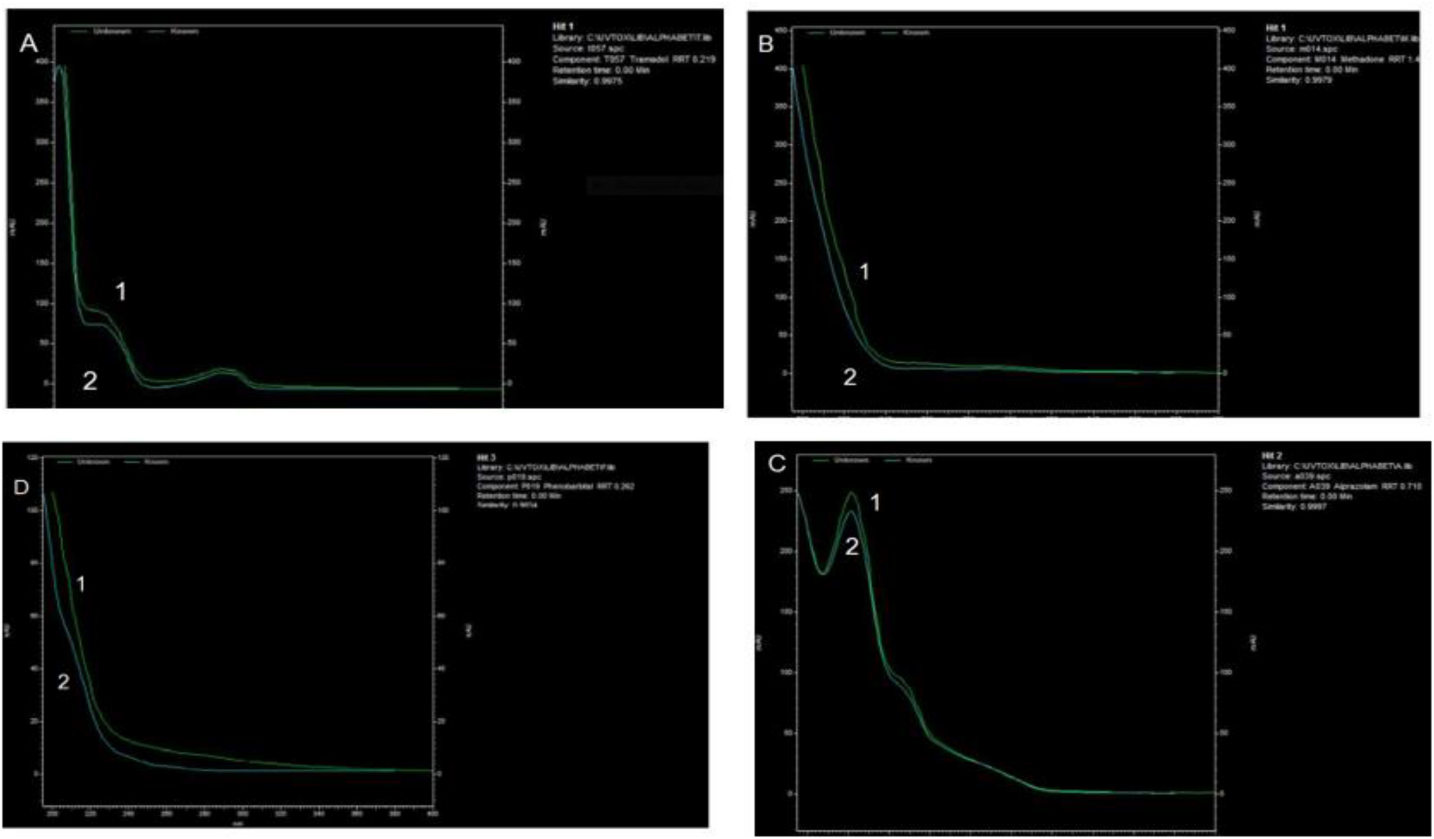
HPLC chromatogram from absorbance measured with diode-array detector (DAD). The 3D plot from HPLC-DAD allowed the peak for tramadol, methadone, phenobarbital, and alprazolam to be identified in the urine sample of a multidrug user case. 1). Standard and 2). Urine spiked with: **(A)** Tramadol **(B)** Methadone **(C)** Alprazolam, and **(D)** Phenobarbital. The extracts were analyzed using HPLC (KNAUER GmbH, Berlin, Germany), C18 column, flow: 1 ml/min, Run time: 40 min and detector type: DAD (Smartline 2800).

In this study, we investigated the prevalence among the GSTT1 and GSTM1 genes and addiction to opioids and methamphetamine. A total of 160 cases (including 80 opium users, 30 methamphetamine users, and 80 healthy individuals as a control group) participated in the study. Overall, all samples were screened using the Acon rapid test, TLC, HPLC, and GC-MS. Positive samples for drugs other than opium alkaloids and methamphetamine were excluded (**Figure 1**). This was done to eliminate potential false positive and negative results caused by sample impurities.

However, a statistically significant association was observed between the GSTM1 gene and morphine addiction under a recessive genetic model. The reference group was pooled n/p and p/p genotypes, and the odds ratio (OR) was 2.15, with a 95% confidence interval (CI) of 1.05-4.39 and a P-value of 0.03. This indicates that individuals carrying two copies of the GSTM1 null allele have a higher risk of developing morphine addiction compared to those carrying at least one copy of the functional allele (**Table 2**). In contrast, there was no statistically significant correlation between the GSTT1 gene genetic variation and morphine or methamphetamine addiction. The OR for the association between morphine addiction and GSTT1 was 1.37, with a 95% CI of 0.58-3.19 and a P-value of 0.46, while the OR for the association between methamphetamine addiction and GSTT1 was 0.91, with a 95% CI of 0.31-2.67 and a P-value of 0.86 (**Table 2**).

**Table 2:** Association between GSTM1 and GSTT1 genes phenotype and addiction to opioids and methamphetamine in the Iranian population.

Gene	Phenotype	Control	Addiction Groups					
			Methamphetamine			Morphine		
		Frequency (%) N=80	Frequency (%) N=30	OR (95% CI)	P-value	Frequency (%) N=50	OR	P-value
GSTM1	Present	49/80 (61.3%)	19/30 (63.2%)	0.92 (0.38-2.18)	0.86	21/50 (42%)	2.15 (1.05-4.39)	0.03*
	Null	31/80 (38.7%)	11/30 (36.7%)			29/50 (59%)		
GSTT1	Present	65/80 (81.3%)	25/30 (83.3%)	0.91 (0.31-2.67)	0.86	38/50 (76%)	1.37 (0.58-3.19)	0.46
	Null	15/80 (18.7%)	5/30 (16.7%)			12/50 (24%)		

OR, Odds Ratio, CI, Confidence Interval.

*P < 0.05: statistically significant.

The results revealed no significant relationship between GSTT1 and GSTM1 allele frequency and morphine and methamphetamine addiction when categorized as carriers and non-carriers of the risk allele (null allele) (**Table 3**).

**Table 3:** Odd ratios for addiction to opioids and methamphetamine in the Iranian population by GSTM1 and GSTT1 genotype.

Gene	Genotype	Addiction Groups			
		Methamphetamine		Morphine	
		OR (95% CI)	P-value	OR (95% CI)	P-value
GSTM1	P/P	Ref	–	Ref	–
	N/P	0.78 (0.24-2.56)	0.69	1.57 (0.42-5.90)	0.49
	N/N	0.76 (0.22-2.58)	0.66	3.07 (0.84-11.26)	0.09
GSTT1	P/P	Ref	–	Ref	–
	N/P	0.99 (0.39-2.49)	0.98	1.26 (0.55-2.88)	0.57
	N/N	0.89 (0.29-2.99)	0.85	1.58 (0.59-4.27)	0.36

P, Present; N, Null; OR, Odds Ratio; CI, Confidence Interval.

## 4 Discussion

The results of the association study between the null allele of GSTM1 and GSTT1 genes, which result in the absence of enzyme activity and addiction to opioids and methamphetamine, suggest that there might be some underlying mechanisms linking these genes to addiction susceptibility (10). These findings are also in line with evidence from literature implying that certain variants of these genes are associated with increased risk for addiction to various drugs, including morphine and methamphetamine. A study explored the association between the GST genetic polymorphisms and methamphetamine abusers in the Japanese population and found that individuals with the GSTP1 Ile105Val variant had a significantly higher risk of methamphetamine abuse compared to individuals with the wild-type genotype (10), which is consistent with the broader literature on genetic risk factors for substance abuse, implying that genetic variation may influence susceptibility to addiction (25). For instance, the study leveraging genome-wide data to investigate differences between opioid use vs. opioid dependence in 41,176 individuals from the Psychiatric Genomics Consortium identified several genetic loci associated with opioid dependence, including some genes involved in the regulation of neuronal development, synaptic function, and inflammation (26). These findings suggest that genetic factors, including GSTM1 and GSTT1 genes, could influence the development of addiction by affecting these underlying biological processes. Actually, recent studies have shown that astrocytes play a crucial role in regulating drug addiction (25). Astrocytes are the most abundant cell type in the brain and have been found to modulate neuronal activity, synaptic plasticity, and neurotransmitter release, thereby influencing drug-related behaviors (27). It has been suggested that impaired astrocytic function could lead to increased susceptibility to neurotoxicity and contribute to the development of addiction (25).

It has been reported that astrocytes express various receptors for neurotransmitters and neuromodulators involved in drug addiction, such as dopamine, glutamate, adenosine receptors, and opioid receptors, including mu (MOR), delta (DOR), and kappa (KOR) receptors (28). Studies have shown that activation of opioid receptors on astrocytes can modulate various cellular processes, such as cytokine release, calcium signaling, and glutamate uptake, which can affect neuronal function and contribute to pain processing and opioid addiction (29). However, the precise role of astrocytic opioid receptors in these processes is still an active area of research. it has been proposed that the activation of these receptors can lead to an increase in intracellular calcium levels, resulting in the release of gliotransmitters, such as glutamate and ATP, which can modulate synaptic transmission and plasticity (30).

Modulation of opioid tolerance and dependence by astrocytes might also be done through the release of inflammatory mediators. Inflammatory mediators, such as tumor necrosis factor-alpha (TNF-α) and interleukin-1 beta (IL-1β), can induce opioid receptor desensitization, leading to decreased opioid sensitivity and increased tolerance (31). Moreover, astrocytes can also release neurotrophic factors, such as brain-derived neurotrophic factor (BDNF), which can promote dopaminergic neuron survival and function, potentially protecting against the neurotoxic effects of opioids (27).

Furthermore, astrocytes have been found to play a role in the metabolism of drugs, including opioids and methamphetamine (32). The GSTT1 and GSTM1 genes are involved in the detoxification and metabolism of drugs, and the presence or absence of functional protein products of these genes may influence the metabolic activity of astrocytes (33). Therefore, the observed association between GSTM1 gene and addiction to morphine may possibly be due to alterations in astrocyte function and their role in modulating drug-related behaviors (34). Further research studies are needed to fully elucidate the mechanisms underlying this association.

### 4.1 Limitations

However, there are a few caveats that should be considered when interpreting the results of this association study: A) Limited sample size: The study had a relatively small sample size, which may limit the generalizability of the findings. A larger sample size would provide greater statistical power and improve the reliability of the results. B) Presence of possible confounding variables: The study did not report on potential confounding variables, such as demographic or environmental factors, that could influence the association between the GSTM1 and GSTT1 genes and addiction to opioids and methamphetamine. C) The study was also retrospective in nature, meaning that the participants were selected based on their addiction status. This may introduce selection bias and limit the ability to draw causal conclusions. Overall, additional studies are required to confirm and generalize the findings while the study provides an evidence of a potential association between the GSTM1 gene and morphine addiction.

## 5 Conclusion

The findings demonstrated that the GSTM1 gene may play a role in the development of morphine addiction; however, further studies with larger sample sizes are warranted to verify the findings and assess the underlying molecular mechanisms. Hence, we highlight the importance of replicating such studies and mechanistic researches in identifying other polymorphisms or genetic pathways that may be involved in addiction.

## Data Availability

The original contributions presented in the study are included in the article/supplementary material. Further inquiries can be directed to the corresponding author.
